# Interference in Bacterial Quorum Sensing: A Biopharmaceutical Perspective

**DOI:** 10.3389/fphar.2018.00203

**Published:** 2018-03-07

**Authors:** Benjamin Rémy, Sonia Mion, Laure Plener, Mikael Elias, Eric Chabrière, David Daudé

**Affiliations:** ^1^IRD, APHM, MEPHI, IHU-Méditerranée Infection, Aix Marseille Université, Marseille, France; ^2^Gene&GreenTK, Marseille, France; ^3^Department of Biochemistry, Molecular Biology and Biophysics, Biotechnology Institute, University of Minnesota, St. Paul, MN, United States

**Keywords:** quorum sensing (QS), bacterial virulence, biofilm, quorum sensing inhibitors, quorum quenching enzymes, antibioresistance, phage resistance, medical devices

## Abstract

Numerous bacteria utilize molecular communication systems referred to as quorum sensing (QS) to synchronize the expression of certain genes regulating, among other aspects, the expression of virulence factors and the synthesis of biofilm. To achieve this process, bacteria use signaling molecules, known as autoinducers (AIs), as chemical messengers to share information. Naturally occurring strategies that interfere with bacterial signaling have been extensively studied in recent years, examining their potential to control bacteria. To interfere with QS, bacteria use quorum sensing inhibitors (QSIs) to block the action of AIs and quorum quenching (QQ) enzymes to degrade signaling molecules. Recent studies have shown that these strategies are promising routes to decrease bacterial pathogenicity and decrease biofilms, potentially enhancing bacterial susceptibility to antimicrobial agents including antibiotics and bacteriophages. The efficacy of QSIs and QQ enzymes has been demonstrated in various animal models and are now considered in the development of new medical devices against bacterial infections, including dressings, and catheters for enlarging the therapeutic arsenal against bacteria.

## Introduction

**Figure 1:**
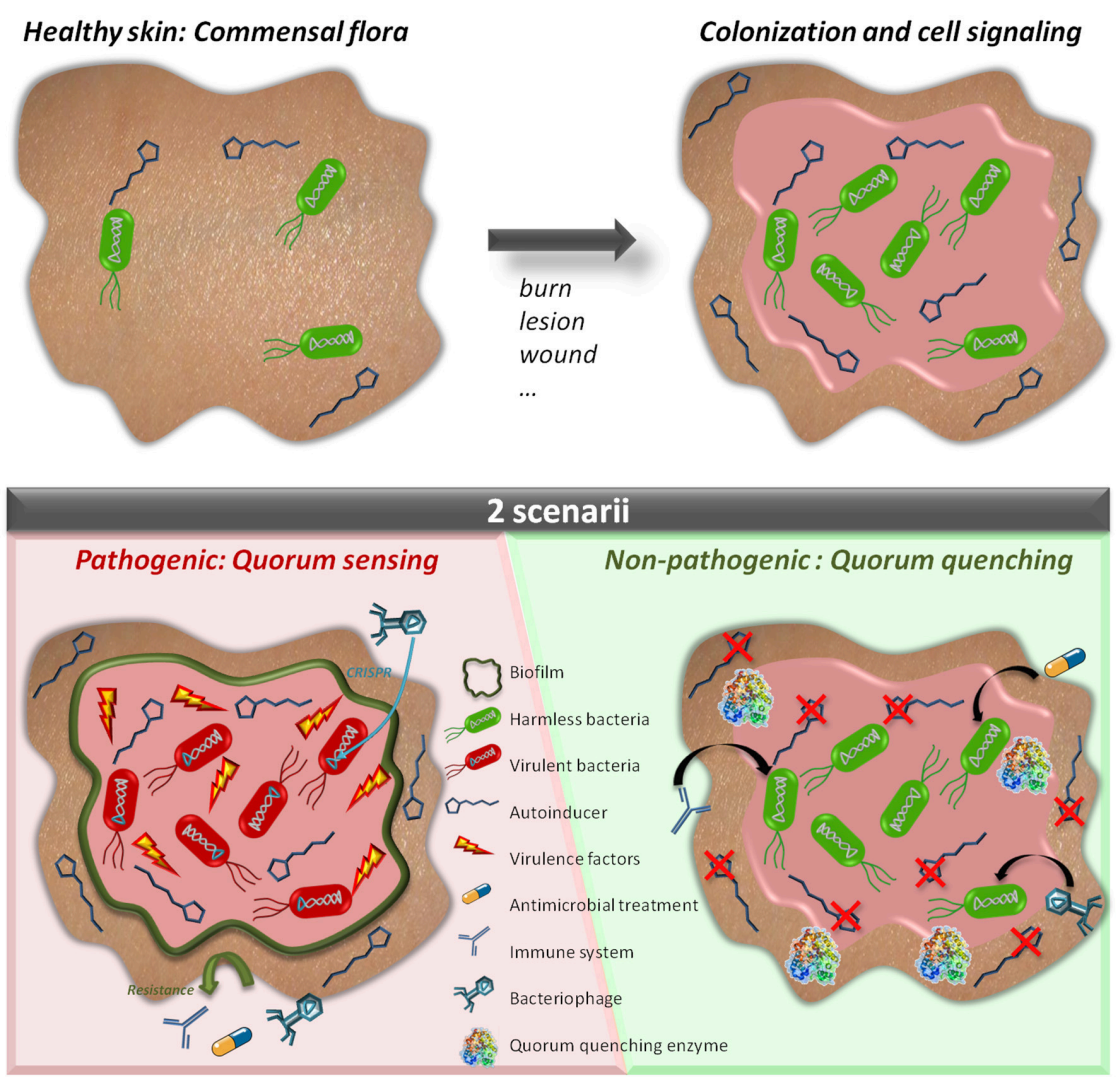
Quorum sensing and quorum quenching in a wounded tissue. The skin usually harbors a natural and commensal flora which is not pathogenic **(Upper Left)**. When a wound or a lesion occurs, bacteria colonize the wounded tissue and further develop being in a favorable environment **(Upper Right)**. While growing, bacteria produce communication molecules (autoinducers). If the molecules are not degraded **(Bottom Left)**, bacteria can synchronize their behavior to secrete virulence factors and produce biofilms which may prevent efficiency of antibiotic or phage therapy. The wound is infected. If the autoinducers are degraded **(Bottom Right)** bacteria do not synchronize their behavior and remain harmless and defenseless. The wound remains colonized but no infection occurs.

**Figure 2:**
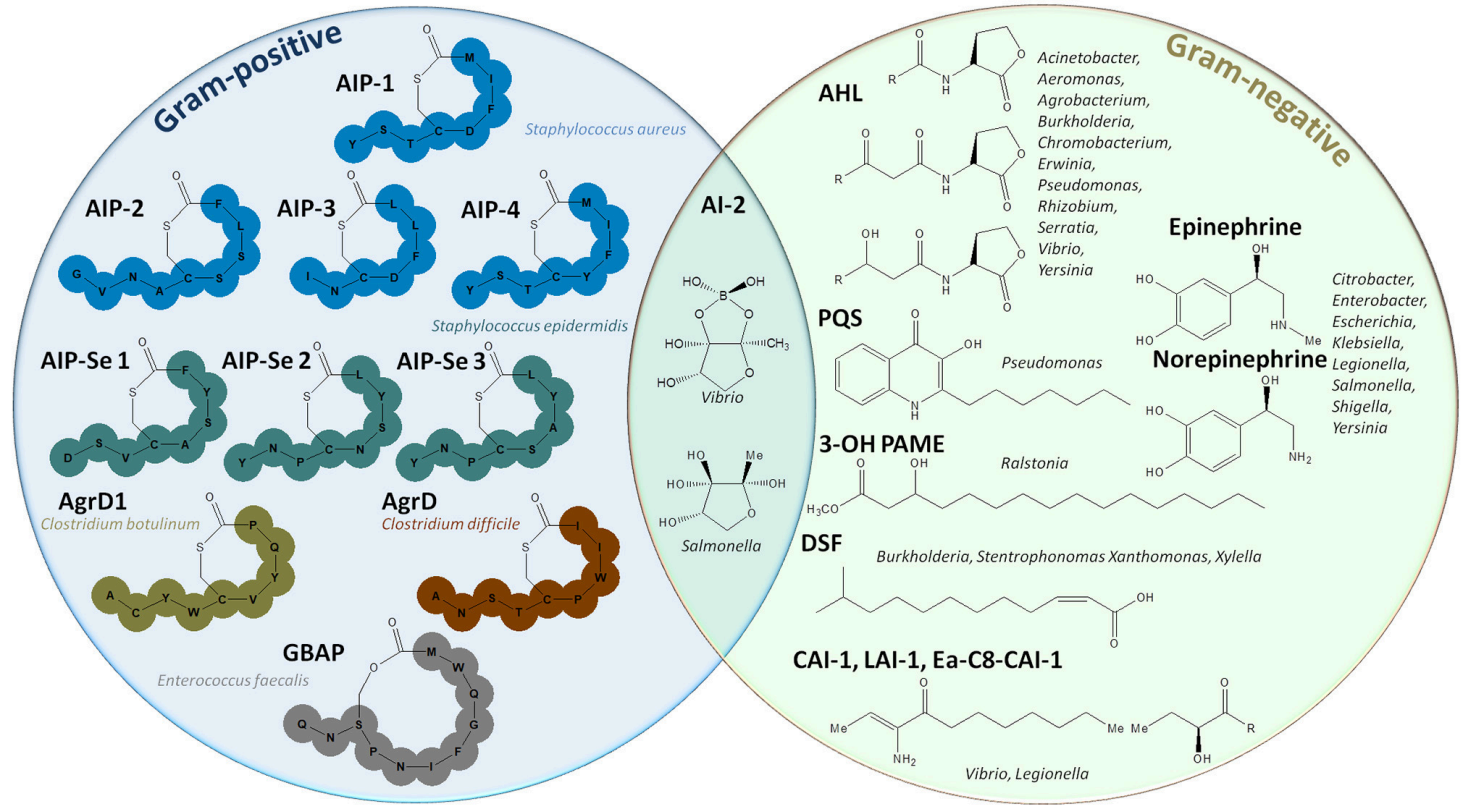
Representation of autoinducer molecules. The left circle represents autoinducing peptides used by Gram-positive bacteria such as *Staphylococcus* spp., *Clostridium* spp., *Enterococcus faecalis* (Monnet et al., 2016). The right circle gives an overview of the different molecules used in Gram-negative quorum sensing: acyl homoserine lactones (AHLs) (Schuster et al., 2013), quinolones (PQS), 4-hydroxypalmitate methyl ester (3-OH-PAME) (Flavier et al., 1997), fatty acids (DSF) (Zhou et al., 2017), epinephrine, and norepinephrine (Kendall and Sperandio, 2007). In the middle, the different forms of AI-2, a furanosyl diester, used by both Gram-positive and Gram-negative bacteria are depicted (Chen et al., 2002).

Quorum sensing (QS) is a molecular mechanism by which bacteria communicate to collectively adapt their behavior according to cell density and the surrounding environment (Figure 1). This communication system enables bacteria to undertake processes that are costly and non-effective at low cell density but that become useful for the whole community at high cell density such as virulence factor synthesis, biofilm formation, and protease and siderophore production (Heilmann et al., 2015). QS consists in the production and sensing of small extracellular molecules, known as autoinducers (AIs), that are released in proportion to cell density (Papenfort and Bassler, 2016). In Gram-positive bacteria, autoinducing peptides (AIPs) were widely studied and reported to induce QS. AIPs are specific to species and strains and have been described in *Staphylococcus* spp., *Clostridium* spp., or *Enterococcus* spp., among others, AIPs (Figure 2; Monnet et al., 2016). Many Gram-negative bacteria, including *Pseudomonas* spp., *Acinetobacter* spp., or *Burkholderia* spp., were reported to use a different class of autoinducers: the acyl-homoserine lactones (AHLs) (Schuster et al., 2013). AHLs are composed of a lactone ring and an aliphatic acyl chain varying in length and modifications (Schuster et al., 2013). A wide variety of other signaling molecules was also identified (Hawver et al., 2016), including fatty acids used by *Xanthomonas* spp., *Burkholderia* spp.*, Xylella* spp. (Zhou et al., 2017), ketones (*Vibrio* spp. and *Legionella* spp.; Tiaden and Hilbi, 2012), epinephrine, norepinephrine and AI-3 (enterohemorrhagic bacteria; Kendall and Sperandio, 2007) or quinolones (*Pseudomonas aeruginosa;* Heeb et al., 2011). Finally, AI-2, a furanosyl borate diester, is used by both Gram-negative and Gram-positive bacteria (Chen et al., 2002; Figure 2). Most Gram-negative bacteria combine several QS systems to integrate different signals either hierarchically, as *P. aeruginosa* in which four QS systems (*las, rhl, iqs*, and *pqs*) act in a network (Lee and Zhang, 2015), or in parallel, as in *Vibrio harveyi* in which three systems are integrated into one regulatory cascade (Plener et al., 2015).

**Figure 3:**
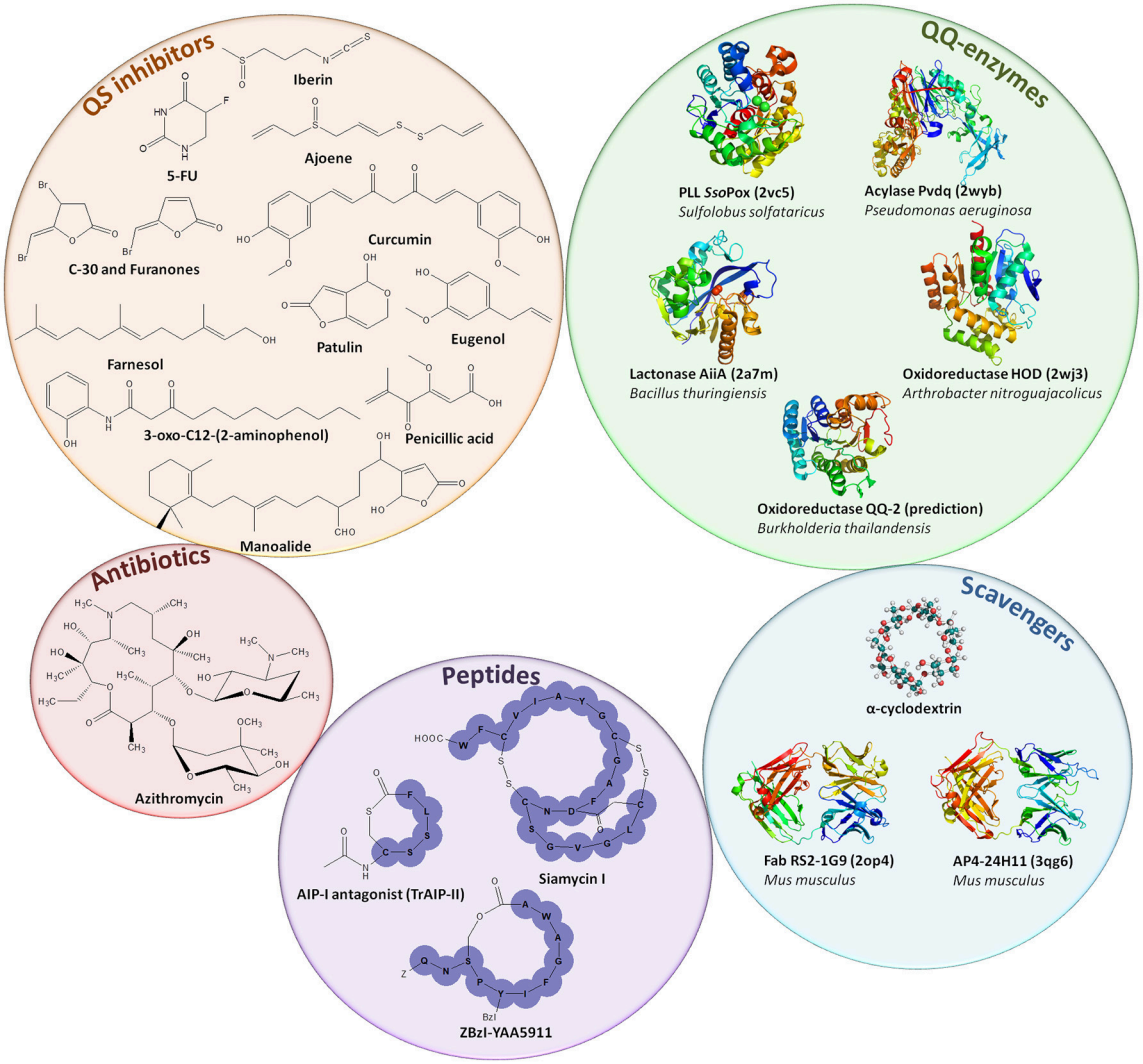
Representation of quorum quenching agents. Quorum sensing inhibitors, mainly acting against AHL or AI-2-based QS, are depicted in the orange circle (Tang and Zhang, 2014). Antibiotics such as azithromycin can be used as QSI at sub-inhibitory concentrations (Swatton et al., 2016). Purple circle represents the QQ peptides used to inhibit Gram-positive QS (Singh et al., 2016). Blue circle represents molecules used to scavenge AIs such as cyclodextrins or derivatives (Morohoshi et al., 2013) and antibodies scavenging AHL (Fab RS2-1G9) or AIP (AP4-24H11) (Park et al., 2007). Green circle depicts QQ enzymes that disrupt AHLs (*Sso*Pox, Pvdq, and AiiA), the quinolone PQS (HOD) and AI-2 signals (QQ-2) (Fetzner, 2015).

Interferences with QS are termed quorum quenching (QQ) (Figure 1). QQ was discovered as a naturally occurring phenomenon first described in 2000 with the identification of a QQ enzyme able to degrade AHL signals from *Erwinia carotovora* (Dong et al., 2000). The enzymatic hydrolysis of AHL led to the disruption of the QS signal. The disruption of bacterial communication can be achieved by several processes: (i) interfering with the production or perception of AIs via small molecules referred to as quorum sensing inhibitors (QSIs) (Tang and Zhang, 2014), (ii) scavenging of AIs by quorum quenching antibodies (Park et al., 2007), and macromolecules such as cyclodextrins (Kato et al., 2006, 2007; Morohoshi et al., 2013), or (iii) by extracellular hydrolysis of the AIs using QQ enzymes (Fetzner, 2015; Figure 3). Several antagonist peptides have been identified among natural compounds or designed to quench Gram-positive bacteria and many QSIs, mainly targeting Gram-negative QS and AI-2 mediated QS, have also been reported (Tang and Zhang, 2014; Singh et al., 2016). Such compounds can be natural products, like polyphenols isolated from tea or honey, ajoene from garlic, eugenol from clove or many others produced by marine organisms and fungi (Tang and Zhang, 2014; Delago et al., 2016), or they can be synthetic, such as 5-fluorouracil (5-FU) or azithromycin (Ueda et al., 2009; Swatton et al., 2016). Many QQ enzymes and macromolecules (Amara et al., 2011; Fetzner, 2015) as well as natural or synthetic QSIs (Dembitsky et al., 2011; Galloway et al., 2011; Stevens et al., 2011; Kalia, 2013; Delago et al., 2016) have been reported to date and exhaustively reviewed. Patents associated with these compounds (Pan and Ren, 2009; Romero et al., 2012; Jiang and Li, 2013) as well as routes to access novel molecules (Scutera et al., 2014) have also been discussed. The mechanisms used by the different QSIs are not always known and most probably differ from one QSI to another (Defoirdt et al., 2013). Some molecules inhibiting QS such as azithromycin are also considered as antibiotics as they can inhibit bacterial growth above a certain concentration (Nalca et al., 2006).

Currently identified QQ enzymes mainly target AHLs and AI-2 mediated QS: phosphotriesterase-like lactonases (PLLs), lactonases, acylases, and oxidoreductases degrade AHL signals (Fetzner, 2015) and oxidoreductases target AI-2 (Dong et al., 2000; Weiland-Bräuer et al., 2016). As QS induces noxious traits such as biofilm formation or virulence, the disruption of bacterial communication appears as a promising strategy to prevent bacteria from synchronizing their virulent behavior. Therefore, QQ approaches may have applications in numerous fields such as agronomy, water engineering, and the marine industry and is particularly relevant in health care (Bzdrenga et al., 2017).

In the current context of the rise of antibiotic tolerance and resistance, novel therapeutic approaches are needed (Kaye and Pogue, 2015). The ability of QQ approaches to inhibit bacterial virulence and biofilm is appealing as this latter is associated with increased antibiotic tolerance (Stewart and William Costerton, 2001). Biofilm formation is triggered via QS and consists in a heterogeneous multi-cellular structure attached to a solid surface, embedded in an extracellular matrix (de la Fuente-Núñez et al., 2013). The extracellular matrix, made of polysaccharides, proteins and extracellular DNA, may prevent some antibiotics from successfully penetrating the cells, inducing antibiotic tolerance (Otto, 2006). The bacterial cells embedded in the matrix also have a slower growth rate and an altered metabolism which further reduces antibiotic efficiency (Olsen, 2015). In addition, biofilm environments combine high cell density and high selection pressure increasing the rate with which resistant cells appear through mutations or gene transfer (Driffield et al., 2008). Biofilms also shelter persistent cells, a non-inheritable trait denoting a subpart of cells present in any bacterial population that will survive antibiotic treatment as a result of being in a different physiological state at the time of the treatment (Brauner et al., 2016). Bacteria in biofilms are considered to be 100–1,000 times more tolerant to antimicrobial compounds as compared to their planktonic lifestyle (Olsen, 2015). Biofilms can also be at the source of infections: it is considered that between 65 and 80% of infections are biofilm associated infections, either by directly infecting the tissue such as lung infection in the case of cystic fibrosis or via a contaminated device such as a catheter (Lebeaux et al., 2013). Hospital-acquired infections (HAIs) affect between 6 and 10% of health care patients in developed countries, the most frequent type of infections being urinary tract infections (Klevens et al., 2007; Lobdell et al., 2012). Eliminating biofilms in health care devices and environment is a challenge to limit and treat HAIs. It is, therefore, essential to develop alternative or complementary treatments to conventional antimicrobial and antibiotic products. To this end, QQ and phage therapy are increasingly studied (Kostakioti et al., 2013; Rémy et al., 2016a).

This review highlights the latest findings and biopharmaceutical perspectives of QQ as well as its potential complementarity with antimicrobial agents, antibiotics and bacteriophages. The eukaryotic models used to prove the efficiency of QQ as a successful anti-virulence and anti-biofilm strategy and the medical applications with QQ devices are also summarized.

## Quorum sensing and the sensitivity to antimicrobial agents

As QS involves a global change in bacterial gene expression and cell physiology, the relationship between QS and antibiotic tolerance is multi-faceted. For example, the addition of AHLs to a logarithmic culture of *P. aeruginosa* was shown to increase the number of persister cells in the population after treatment with carbenicillin and ciprofloxacin (Möker et al., 2010). Furthermore, transcriptomic analysis with the QS transcription regulator MvfR (PqsR) in *P. aeruginosa* PA14 revealed that QS induces the expression of peroxidases which provide protection against reactive oxygen species (H_2_O_2_) and β-lactam antibiotics (Maura et al., 2016). In another study using *P. aeruginosa* PAO1, VqsM, a global regulator that induces QS, was shown to mediate antibiotic tolerance by inducing the expression of *nfxB*, an antibiotic resistance regulator, providing increased tolerance to quinolones, tetracycline, and kanamycin via regulation of *mexC-mexD-oprJ* operon expression (Poole et al., 1996; Liang et al., 2014).

Although some physiological aspects may be involved in QS-mediated tolerance to antibiotics, many reports focus on the importance of biofilm in antibiotic tolerance of bacteria (Høiby et al., 2010), causing many difficulties for treatments of clinical infections (Høiby et al., 2011). Those effects have been frequently observed with *P. aeruginosa* (Strateva and Yordanov, 2009) in both model and clinical strains (Hill et al., 2005) as well as in other species such as *Klebsiella pneumoniae* (Vuotto et al., 2014, 2017) and *Staphylococcus aureus* (Savage et al., 2013). The particular conditions that the biofilm mode of growth provides to bacteria favors the development of different defense mechanisms and phenotypes: physical barrier, modification of gene expression, and cellular physiological states (e.g., persister cell) (de la Fuente-Núñez et al., 2013). In *P. aeruginosa*, the *rhl*, and *las* QS systems are essential for biofilm formation and their disruption is correlated with a higher sensitivity to the host immune system and antimicrobial compounds (Davies et al., 1998; Shih and Huang, 2002; Bjarnsholt, 2005b). Moreover in *P. aeruginosa*, another QS system, the *pqs* system, has been demonstrated to mediate a programmed cell death inducing extracellular DNA release which promotes biofilm formation and antibiotic tolerance, benefitting the rest of the cell population (Hazan et al., 2016). In clinical isolates of *Acinetobacter baumannii*, the presence of levofloxacin or meropenem antibiotics was reported to induce the overexpression of an efflux pump which stimulates the release of AHL and thus enhances the formation of QS-mediated biofilm, increasing antibiotic tolerance (He et al., 2015).

Regarding the important role of QS in biofilm formation and antibiotic tolerance, combination therapy with QQ was investigated. In *P. aeruginosa*, the use of a pharmacological compound, benzamide-benzimidazole, inhibiting the QS regulator MvfR (PqsR) decreased biofilm formation and restored antibiotic susceptibility (Starkey et al., 2014; Maura and Rahme, 2017). Baicalin hydrate and hamamelitannin, an AHL-targeting QSI and a peptide-based QSI, enhanced biofilm disruption in both Gram-negative (*P. aeruginosa* and *Burkholderia cepacia* complex) and Gram-positive (*S. aureus*) bacteria and showed synergistic effects in cotreatment with tobramycin and clindamycin or vancomycin respectively both *in vitro* and *in vivo* (Brackman et al., 2011b). From aminoglycosides (Jakobsen et al., 2012; Stenvang et al., 2016) to quinolones (Guo et al., 2016), polypeptides (Furiga et al., 2016; Bulman et al., 2017), cephalosporins (Maura and Rahme, 2017), and glycopeptides (Das et al., 2016), the efficiency of a large range of antibiotics is enhanced by the addition of QSIs.

Taken together these results suggest that using QSIs is a potential way of increasing antibiotic sensitivity and thereby lower antibiotic active doses. Additionally, similar trend and efficiency have also been observed with a lactonase QQ enzyme and the antibiotic ciprofloxacin in a mice model (Gupta et al., 2015). Combining antimicrobial agents and QQ was showed to have encouraging synergistic effects, highlighting that QQ is a good strategy to decrease antibiotics use and fight against the increasing problem of antibiotic resistance. Nevertheless, if QQ can help to prevent and treat infections, it cannot be used on its own to treat acute infections by antibiotic resistant strains.

## Relationship between quorum sensing and the sensitivity to bacteriophages

Recently, interest has considerably increased in phage therapy as a way of treating infections caused by multi-drug resistant bacteria (Pires et al., 2017). Bacteriophages are the most abundant bacterial predators on the planet and they are still used to treat bacterial infections in Eastern Europe (Brüssow and Hendrix, 2002). Although bacteriophages represent an interesting solution to circumvent antibiotic resistance, bacteria have also developed resistance mechanisms to counteract phage actions (Labrie et al., 2010). Firstly, phage entry can be decreased by extracellular matrix production, or by modifying the phage receptor structure or expression (Chapman-McQuiston and Wu, 2008; Labrie et al., 2010). Once inside the cell, phage DNA can be recognized and degraded by restriction enzymes or the adaptive inducible CRISPR-Cas (clustered regularly interspaced short palindromic repeat and CRISPR associated proteins) system (Barrangou et al., 2007; Labrie et al., 2010). Other potential metabolism adaptations could also provide bacteriophage resistance (Qin et al., 2017). As the risk of phage infection rises with cell density (Abedon, 2012), QS-mediated resistance would provide protection during high risk conditions while limiting the overall fitness cost of those mechanisms (Hall et al., 2011).

The relationship between QS and bacteriophage sensitivity was originally observed in *P. aeruginosa* (Glessner et al., 1999), but the ability of QS to regulate phage defense mechanisms in *Escherichia coli* was only demonstrated years later (Høyland-Kroghsbo et al., 2013). The authors showed that AHLs induced a reduction in the phages lambda and chi adsorption rate by reducing the number of receptors at the cell surface. In *Vibrio cholerae*, a deficiency in AI synthase genes, and thereby in QS induction, reduced phage resistance which could be restored by the addition of exogenous AIs, AI-2, and CAI-1 (Hoque et al., 2016). This increased phage resistance upon QS activation was explained by the downregulation of O-antigen synthesis which decreased phage adsorption and by an increase in hemagglutinin protease production which was shown to inactivate phages (Hoque et al., 2016). Similarly, the addition of synthetic AHLs to a *Vibrio anguillarum* QS deficient strain improved phage resistance (Tan et al., 2015). Indeed, AHL production was negatively correlated with phage receptor *ompK* expression. Recently, it was shown in *Serratia marcescens* that the CRISPR-Cas immune system was also under QS regulation (Patterson et al., 2016). Both the acquisition of immunity and DNA degradation mechanism coordinated by this system were negatively impacted by the absence of QS signal in a synthase mutant. Besides, by analyzing former datasets (Bowden et al., 2013; Gao et al., 2015), the authors suggested that this type of regulation might occur in *Pectobacterium atrosepticum* as well as in *Burkholderia glumae*. Similar QS control of CRISPR-Cas system was demonstrated in *P. aeruginosa* PA14 in which the expression of the CRISPR-Cas genes is down-regulated in a strain deleted for both AI synthase genes *lasI* and *rhlI* (Høyland-Kroghsbo et al., 2017). To evaluate whether QQ would increase phage sensitivity, QSI effects were investigated on resistance mechanisms and on phage susceptibility phenotypes. The use of penicillic acid increased *P. aeruginosa* sensitivity to phages by increasing the proportion of sensitive viable cells in the population (Qin et al., 2017). Finally, the QSI baicalin was shown to inhibit the QS stimulation of CRISPR-Cas system in *P. aeruginosa* which could prevent the use of this adaptive system by bacteria in case of phage infection (Høyland-Kroghsbo et al., 2017).

In light of these findings, the use of QQ compounds is a highly promising way to develop new therapeutic applications. Indeed, their use in combination with phage therapy treatments could increase bacterial sensitivity to phages by synergistic effects. In addition, disturbing the QS of one species was demonstrated to induce a reduction in total biomass in multimicrobial cultures under phage infection, leading to the consideration that QQ combined with phage therapy could as well be efficient against polymicrobial infections (Mumford and Friman, 2017). To prove the efficiency of QQ as antivirulent agent proper *in vivo* assays and proof of concepts on animal models should be performed.

## Antivirulence activity of quorum quenchers *in vivo*

In order to evaluate the role of QS in pathogenicity several models have been developed over the past few years. Three models, from a simple unicellular model to complex models, commonly used to assess the benefits of QQ and studies conducted on humans are summarized below.

## Amoebal infection models

Free-living amoebae are eukaryotic organisms found either in a resting (cyst) or a vegetative (trophozoite) form feeding on bacteria among other organisms (algae or fungi). In this way, they use phagocytosis coupled with lysosomal digestion which is close to macrophage bacterial elimination pathway (Greub and Raoult, 2004; Matz and Kjelleberg, 2005; Hilbi et al., 2007). Considering these close interactions, amoeba were considered to test bacterial production of virulence factors (Cosson et al., 2002; Clamens et al., 2017), biofilm (Matz et al., 2004, 2005), and secretion systems (Pukatzki et al., 2002, 2006; Matz et al., 2008). Classically, the evaluation of bacterial virulence in amoebae relies on the capacity of amoebae to grow or not to grow in the presence of pathogenic bacteria (Cosson et al., 2002; Pukatzki et al., 2002). The link between virulence factors and QS in different bacterial species was evaluated using this approach and was extensively described for *P. aeruginosa*. QS-deficient mutants of *P. aeruginosa* had a decreased virulence toward the amoeba *Dictyostelium discoideum* (Cosson et al., 2002; Pukatzki et al., 2002). Although this model is fast and convenient for large scale experiments such as screening assays, the use of this model is limited, as both culture conditions and amoeba species may strongly affect the results (Weitere et al., 2005). Despite these limitations the amoeba model was recently used to test a QQ enzyme based on the well-characterized assay with *P. aeruginosa* and *D. discoideum* (Clamens et al., 2017). The over-production of *P. aeruginosa* PA14 aliphatic amidase AmiE resulted in a disruption of QS and reduction of virulence in a *D. discoideum* plate killing assay (Clamens et al., 2017).

## *Caenorhabditis elegans* infection models

The roundworm *Caenorhabditis elegans* is a widely used multicellular organism model to study microbial virulence (Tan et al., 1999b; Garsin et al., 2001; Alegado et al., 2003; Ermolaeva and Schumacher, 2014). Like amoebae, *C. elegans* is a convenient model for high throughput evaluation of QS impact on bacterial virulence (Tan et al., 1999b; Garsin et al., 2001; Rasmussen et al., 2005; O'Loughlin et al., 2013). However, in contrast to amoebae, *C. elegans* has an innate immune system which results in a closer comparison with the human immune response (Ermolaeva and Schumacher, 2014) and is particularly relevant for studying pathogenicity. Classically, *C. elegans* is fed using the bacteria of interest and the survival rate is followed. Two types of assays can be performed: (i) a fast killing assay which leads to worm death in a few hours to assess the presence of toxins, and (ii) a slow killing assay with death occurring after several days to evaluate bacterial colonization (Tan et al., 1999a; Köthe et al., 2003; Park et al., 2017).

In order to decipher the importance of QS in virulence, many experiments were dedicated to study the pathogenicity of bacterial mutants impaired in AI synthesis or perception. QS inactivation in different *P. aeruginosa* strains resulted in a decrease in worm mortality (Darby et al., 1999; Tan et al., 1999b; O'Loughlin et al., 2013; Mukherjee et al., 2017). The *C. elegans* model was also used to show the link between QS and virulence of other various Gram-negative bacteria including *Chromobacterium violaceum* (Swem et al., 2009), *E. coli* (Lee et al., 2011), *Yersinia pseudotuberculosis* (Atkinson et al., 2011), *B. cepacia* (Köthe et al., 2003), *Burkholderia cenocepacia* (Deng et al., 2012), or *Burkholderia pseudomallei* (Song et al., 2005). Moreover, the link between QS and pathogenicity was also shown for Gram-positive bacteria such as *Enterococcus faecalis* (Garsin et al., 2001; Sifri et al., 2002) and *S. aureus* (Sifri et al., 2003). Considered as a whole, these studies highlight that QS triggers virulence in many bacteria.

In addition to genetic mutations, the roundworm model was used, alongside traditional *in vitro* tests, to prove the efficiency of QSIs as well as QQ enzymes or bacteria (Table 1). Though the impact on survival may vary according to the assay used and the culture conditions, all the QQ agents tested were shown to efficiently decrease virulence in both Gram-positive and negative bacteria and thus enhancing *C. elegans* survival up to 100% notably with the QQ enzyme BpiB09 targeting AHLs (Bijtenhoorn et al., 2011). The QSI having the more drastic effect on *C. elegans* survival after infection with *P. aeruginosa* PAO1 is 4-nitro-pyridine-N-oxide, a non-toxic chemical compound, which almost fully restored worm survival (Rasmussen et al., 2005). The most efficient natural QSIs are extracts from *Conocarpus, Callistemon vinimalis*, or *Bucida buceras* with a restoration of survival up to 87% (Adonizio et al., 2008; Table 1). Moreover, a synergistic effect with antibiotics was reported for the QSIs baicalin and hamamelitannin (Brackman et al., 2011b).

**Table 1:** *C. elegans* survival rate upon quorum quenching of several virulent bacteria.

**Bacteria**	**Strain**	**QQ agent (concentration)**	Survival rate QQ/control (Time)^*^	**References**
**QUORUM SENSING INHIBITORS (QSI)**				
*B. cepacia*	LMG16656 LMG18828	Baicalin hydrate (100 μM)	≈50/≈25% (48 h) ≈35/≈15% (48 h)	Brackman et al., 2011b
*C. violaceum*	ATCC31532	Chloro lactone (20 μM)	100/0% (48 h)	Swem et al., 2009
*E. coli*	O157:H7	Broccoli extract (0,5% v/v)	50/21,5% (8 days)	Lee et al., 2011
*P. aeruginosa*	PAO1	4-nitro-pyridine-N-oxide (100 μM)	95/0% (5 h)	Rasmussen et al., 2005
		Garlic extract (2% v/v)	60/0% (5 h)	Rasmussen et al., 2005
		Extract from *Conocarpus, Callistemon viminalis* or *Bucida buceras* (1 mg/mL)	84–87/0% (4 h)	Adonizio et al., 2008
		Curcumin (3 μg/mL)	28/0% (100 h)	Rudrappa and Bais, 2008
		2,5-piperazinedione (100 μg/mL)	66/0% (84 h)	Musthafa et al., 2012a
		Phenylacetic acid (200 μg/mL)	53/0% (84 h)	Musthafa et al., 2012b
		Clove oil (1,6% v/v)	62/0% (96 h)	Husain et al., 2013
		Fractionated methanol extract of *Terminalia chebula Retz*. (0,5 mg/mL)	50/0% (72 h)	Sarabhai et al., 2013
		Menthol (800 μg/mL)	58/0% (96 h)	Husain et al., 2015a
		Methanol extract of *Trigonella foenum-graceum* (1 mg/mL)	48/0% (96 h)	Husain et al., 2015b
		Oleanolic aldehyde coumarate (200 μM)	48/20% (4 h)	Rasamiravaka et al., 2015
		*Mangifera indica* methanol leaf extract (800 μg/mL)	72/0% (4 8 h)	Husain et al., 2017
	PAO1 ATCC9027	Baicalin hydrate (100 μM)	≈30/≈10% (48 h) ≈50/≈25% (48 h)	Brackman et al., 2011b
	PA14	Extract from *Conocarpus, Callistemon viminalis* or *Bucida buceras* (1 mg/mL)	53–90/0% (4 h) 57–60/0% (58 h)	Adonizio et al., 2008
		Meta-bromo-thiolactone (50 μM)	77/≈20% (24 h)	O'Loughlin et al., 2013
	Pa1 (clinical isolate)	Tea polyphenols (3,125 mg/mL)	63/20% (48 h)	Yin et al., 2015
*S. aureus*	Mu50	Hamamelitannin (250 μM)	≈55/≈15% (48 h)	Brackman et al., 2011b
*V. anguillarum*	LMG441	3,4-dichloro-cinnamaldehyde (10 μM)	≈90/71% (48 h)	Brackman et al., 2011a
*V. harveyi*	BB120	3,4-dichloro-cinnamaldehyde (10 μM)	≈80/49% (48 h)	Brackman et al., 2011a
*Vibrio vulnificus*	LMG16867	3,4-dichloro-cinnamaldehyde (20 μM)	≈80/15% (48 h)	Brackman et al., 2011a
**QUORUM QUENCHING ENZYMES**				
*B. cepacia* complex	46 strains	AiiA, lactonase from *Bacillus sp*. 240B1	100/0–100% (5 days)^**^	Wopperer et al., 2006
*P. aeruginosa*	PAO1	AiiD, acylase from *Ralstonia* strain XJ12B	≈85/5% (4 h)	Lin et al., 2003
		PvdQ, acylase from *P. aeruginosa* PAO1	≈75/0% (4 h) ≈60/≈35% (72 h)	Papaioannou et al., 2009
		BpiB09, short chain dehydrogenase reductase	100/0% (4 h)	Bijtenhoorn et al., 2011
		MomL, lactonase from *Muricauda olearia* Th120 (0,5 U/mL)	≈95/≈50% (24 h) ≈90/≈40% (48 h)	Tang et al., 2015
*Y. pseudotuberculosis*	YpIII	AiiA, lactonase from *Bacillus subtilis*	Reduce biofilm infection severity^***^	Atkinson et al., 2011
**QUORUM QUENCHING BACTERIA**				
*B. cenocepacia*	LGM16656	Rhizosphere, water, mucus or intestines of flounders isolated bacteria	Increased survival (48 h)^***^	Christiaen et al., 2014
*P. aeruginosa*	PAO1	*Pseudomonas, Pseudoalteromonas, Delftia, Arthrobacter, …*	Increased survival (48 h)^***^	Christiaen et al., 2014

* *Survival or not paralyzed at given time*.

** *Estimated from score and strains dependent*.

*** *No survival rate (only increased in survival rate or other)*.

*Caenorhabditis elegans* is a highly valuable invertebrate model enabling high throughput screening (for bacterial mutants or QQ compounds) and gives a very deep insight into virulence regulation, modulation by QQ agents and, in general, by anti-infective molecules (Kong et al., 2016). In most cases, QQ with either QSI or enzymes seems to be able to reduce mortality due to a wide range of bacteria in *C. elegans* and thus gives a relevant proof of concept of QQ as antivirulent agent in a multicellular organism. However, it also has some limitations, such as the living parameters of the worm which differ from bacterial ones (e.g., growth temperature around 20°C), and the physiopathology of the roundworm which is very different from the human one. Furthermore, as for amoebae, the influence of assay conditions on the outcome of the assay have been highlighted by several studies (Mahajan-Miklos et al., 1999; Tan et al., 1999a; Gallagher and Manoil, 2001).

## Murine infection models

Mammalian models, such as rats or mice, are commonly used to decipher the impact of QS in bacterial infections. Indeed, mutations or deletions of QS related genes were shown to reduce the mortality or severity of the infections in the lungs (Pearson et al., 2000; Wu et al., 2001; Lesprit et al., 2003; Sokol, 2003), wound burns (Rumbaugh et al., 1999; Tan et al., 1999b), peritonitis (Sifri et al., 2002), the prostate (Nelson et al., 2009), and the intraperitoneal foreign body model (Christensen et al., 2007), with the exception of *Staphylococcus epidermidis* (Xu et al., 2006). In the vast majority of cases, QQ approaches result in a decrease in mortality, accelerate recovery and reduce bacterial colonization.

In lung infection models, *P. aeruginosa* colonization or related mortality was reduced by furanones (Hentzer et al., 2003; Wu, 2004), sub-minimal inhibitory concentrations of azithromycin (Hoffmann et al., 2007), garlic extract (Bjarnsholt, 2005a), and also by the inhalation of the lactonase *Sso*Pox (Hraiech et al., 2014). In skin wound models, a wide range of QSIs reduced *S. aureus* pathogenicity (Cirioni et al., 2013; Simonetti et al., 2016; Muhs et al., 2017; Todd et al., 2017). Similar inhibition of pathogenicity was observed with an AIP-targeting antibody (Park et al., 2007). Treatment with QS inhibiting peptide strongly reduced *S. epidermidis* colonization in a graft associated infection (Balaban et al., 2003). The efficiency of QQ was also demonstrated in a burn wound infection model with *P. aeruginosa* and an AHL degrading enzyme (Gupta et al., 2015) or PqsR (MvfR) inhibitors (Lesic et al., 2007) and, in an excision injury model, with the use of tea polyphenols as QSIs (Yin et al., 2015). Moreover, the combination effect of QQ molecules and antibiotics *in vivo* was demonstrated against both Gram-positive and negative bacteria. Indeed, for *B. cenocepacia*, the combination of baicalin and tobramycin allowed to reduce lung colonization by 2 and 1 log of CFU compare to control and antibiotic alone respectively (Brackman et al., 2011a). The use of ciprofloxacin and a lactonase to treat *P. aeruginosa* wound burn infection enabled to reduce mortality and global bacterial dissemination to internal mice organs (Gupta et al., 2015). Furthermore, the cotreatment with a QSI and an antibiotic also drastically reduced colonization of artificial foreign body (e.g., catheter or implants) by *S. aureus* (Cirioni et al., 2013; Simonetti et al., 2016), *S. epidermidis* (Balaban et al., 2003), and *P. aeruginosa* (Christensen et al., 2012; Das et al., 2016). Those examples increased the interest of reducing antibiotic tolerance by QQ either in infected organs or medical device associated infections.

Murine models are useful and common tools to investigate the QQ impact on bacterial infections thanks to their adaptive and innate immune systems together with a physiology closely related to human beings. Furthermore, they are usually necessary and required as preclinical tests before starting human trials. At this stage of drug development, QQ seems to demonstrate great efficiency to reduce either morbidity or deleterious impacts for a wide variety of infections. However, murine models are less prone to screening steps because of practical and ethical problems unlike *C. elegans* or amoeba (van der Worp et al., 2010). Furthermore, some physiological aspects of a pathology are not fully mimicked in murine models like wound healing or inflammation (van der Worp et al., 2010; Seok et al., 2013; Abdullahi et al., 2014).

## Clinical trials in humans with quorum sensing inhibitors

So far, only previously approved or commercialized QSIs were used in clinical trials, even if their primary use and approved biological activity did not relate to bacterial QS at all, but rather to their bactericidal, antimicrobial activities (antibiotics) or their cytotoxicity (anti-cancer molecules) (Walz et al., 2010; van Delden et al., 2012).

In the early 2000s, azithromycin (Figure 3) was used in clinical trials to treat cystic fibrosis (Wolter et al., 2002; Saiman et al., 2003) and pulmonary transplanted patients (Gerhardt et al., 2003). This macrolide antibiotic improved patient's quality of life but did not lead to a decrease of bacterial load (Saiman et al., 2003). At the same period, the ability of azithromycin at non bactericidal concentrations to disrupt bacterial signaling in *P. aeruginosa* was demonstrated *in vitro* (Tateda et al., 2001). Later, the impact of azithromycin on *P. aeruginosa* QS in ventilator-associated pneumonia patients was evaluated (van Delden et al., 2012). The authors described beneficial anti-virulence effects of azithromycin in a high-risk group of patients, yet results were not significant enough.

Garlic is also known for its QQ properties (Rasmussen et al., 2005) and was used in a trial to treat cystic fibrosis patients, although, no clear evidence has emerged of the curative effect of garlic extract on patient health (Smyth et al., 2010).

Finally, the anti-cancer drug (Longley et al., 2003), 5-FU, a pyrimidine analog (Figure 3), was demonstrated to inhibit QS-regulated virulence in *P. aeruginosa in vitro* (Ueda et al., 2009) and was further used for the coating of functionalized catheters, which were shown to be efficient during clinical trials (Jacobsen et al., 2008; Walz et al., 2010).

In the end, very few QQ molecules reached human clinical trials but they tend to demonstrate some beneficial effects of QSIs. Although many proofs of concept were performed in animal models, further efforts have to be dedicated to the validation of this approach in clinical phases to confirm its therapeutic relevance.

## Use of quorum quenching molecules in medical devices

Medical devices are involved in numerous HAIs (Neoh et al., 2017). Multi-drug resistant and/or biofilm forming bacteria are mainly responsible for HAIs causing severe medical complications, high morbidity and risk of mortality. Considering the ability of QQ to prevent bacterial virulence (Grandclément et al., 2016), the development of novel medical devices using QQ agents is of outmost interest. New generations of catheters (Mandakhalikar et al., 2016), dressings (Rémy et al., 2016a; Bzdrenga et al., 2017), aerosols (Hraiech et al., 2014), contact lenses (Jain et al., 2016), implantable devices (Francolini et al., 2017), or orthopedic and trauma devices (Moriarty et al., 2016) are currently being developed (Table 2).

**Table 2:** Quorum quenching based medical devices.

**QQ strategy**	**QQ agent**	**Application**	**References**
QSI	5-FU	Catheters	Jacobsen et al., 2008; Walz et al., 2010
	Furanones	Catheters	Hume et al., 2004
	DHP	Coatings	Ozcelik et al., 2017
	TZD-8	Urinary catheters	Shenderovich et al., 2015
	Furanone and DHP derivatives	Implanted medical devices	Taunk et al., 2016
Peptides	TrAIP-II	Colonization-resistant materials	Kim et al., 2017
	Macrocyclic peptides	Nanofiber coatings	Kratochvil et al., 2017
	FS3	Prosthesis	Cirioni et al., 2013
	RIP	Dacron graft	Balaban et al., 2003
QQ Enzymes	PLL *Sso*Pox from *S. solfataricus*	Coatings, membranes, aerosols	Ng et al., 2011; Hraiech et al., 2014; Guendouze et al., 2017
	Acylase from *A. melleus*	Catheters and other coated devices	Ivanova et al., 2015b; Grover et al., 2016
	Acylase from *A. melleus* and α-amylase from *B. amyloliquefaciens*	Catheters	Ivanova et al., 2015a
	Lactonase from *Bacillus* sp. ZA12	Topical treatments	Gupta et al., 2015
	Acylase from porcine kidney	Nanofibers	Lee et al., 2017
	AI-2 processing kinase LsrK	Capsules	Rhoads et al., 2017
Natural compounds	Polyphenols of honey	Nanovectors	Prateeksha et al., 2017

*5-FU, 5-fluorouracil; Furanone, 3-(10-bromohexyl)-5-dibromomethylene-2(5H)-furanone; DHP, 5-methylene-1-(prop-2-enoyl)-4-(2-fluorophenyl)-dihydropyrrol-2-one; TZD-8, Thiazolidinedione-8; TrAIP-II, a truncated autoinducer peptide (AIP-II) with the exocyclic tail replaced by and acetyl group; FS3, RNA-III inhibiting peptide (RIP) analog (YAPWTNF-NH_2_)*.

QSIs were first considered for the functionalization of catheters. Covalently-attached furanones were shown to decrease biofilm formation by *S. epidermidis* ATCC 35984 and to control infection for 65 days in an *in vivo* sheep model (Hume et al., 2004). 5-FU was used to coat a central venous catheter and was demonstrated to be efficient and comparable to classically used chlorhexidine/silver sulfadiazine coated catheters in a clinical study involving 960 adult patients in 25 US intensive care units (Jacobsen et al., 2008; Walz et al., 2010). Although the link to QS was not made by the authors, the 5-FU coated catheters showed reduced contamination levels, by Gram-negative bacteria, as compared to the traditional coating which could be a clue as to interference with AHL dependent QS in this study. Poly(ethylene glycol)-based coating containing the QSI DHP (5-methylene-1-(prop-2-enoyl)-4-(2-fluorophenyl)-dihydropyrrol-2-one) was recently shown to reduce *S. aureus* strain 38 and *P. aeruginosa* MH602 colonization (Ozcelik et al., 2017). Combinations of DHP and furanone derivatives were also covalently attached onto glass surfaces and significantly reduced the adhesion of *S. aureus* SA38 and *P. aeruginosa* PAO1 (Taunk et al., 2016). A delivery system based on varnishes releasing the QSI thiazolidinedione-8 (TZD-8) was used on catheters and were active against *Candida albicans* biofilms (Shenderovich et al., 2015). Notably, honey polyphenols were introduced into a scaffold of selenium nanovectors for quenching *P. aeruginosa* PAO1 *in vitro* and *in vivo* (Prateeksha et al., 2017).

For *agr*-based QS in *S. aureus*, inhibiting peptides were also successfully incorporated into biomaterials. Macrocyclic peptides were loaded into non-woven polymer nanofibers by electrospinning and showed to retain biological activity against *S. aureus* after releasing over a 3 week period (Kratochvil et al., 2017). Click chemistry was also considered for covalently coating surfaces with pro- and anti-QS peptides, AIP-I and TrAIP-II respectively and showed efficacy against *S. aureus* strains (Kim et al., 2017). The synergy of QS inhibiting peptide FS3 with antibiotics was also demonstrated, with daptomycin being highly effective against staphylococcal infections when combined with a FS3-coated prosthesis (Cirioni et al., 2013). Similarly, for several strains of *S. epidermidis*, the RNAIII-Inhibiting peptide (RIP) was efficient in reducing infection when incorporated into a Dacron graft (Balaban et al., 2003).

Although QS inhibiting materials were obtained after covalent immobilization of QSIs or peptides, QQ enzymes were also thoroughly investigated as these compounds, acting on secreted autoinducers, do not need direct contact with the cells to disrupt communication. Acylase from *Aspergillus melleus* was successfully incorporated into polyurethane coatings and silicon catheters reducing biofilm formation of *P. aeruginosa* ATCC 10145 and PAO1 respectively (Ivanova et al., 2015b; Grover et al., 2016). Combination of the acylase with α-amylase from *Bacillus amyloliquefaciens* delayed biofilm development of both *P. aeruginosa* ATCC 10145 and *E. coli* ATCC 25922 for up to 7 days in an *in vivo* rabbit model (Ivanova et al., 2015a). Acylase from porcine kidney was also immobilized on carboxylated polyaniline nanofibers for the development of nanobiocatalysts limiting biofilm formation of *P. aeruginosa* PAO1. Topical treatment involving lactonase from *Bacillus* sp. ZA12 was also investigated in a burn infection model on mice using *P. aeruginosa* PAO1 (Gupta et al., 2015). Application of a lactonase-containing gel after 10^6^ bacteria burn infection prevented systemic spread, decreased mortality, and showed synergistic effect with ciprofloxacin.

Because enzyme stability is a major bottleneck in the development of bio-based materials, catalysts from extremophile environments were considered. Particularly, PLL *Sso*Pox from *Sulfolobus solfataricus* was found to be a highly attractive way of quenching bacterial virulence (Rémy et al., 2016a; Bzdrenga et al., 2017). This highly robust enzyme (Hiblot et al., 2012; Rémy et al., 2016b), was first immobilized onto nanoalumina membranes while retaining strong efficacy for reducing virulence factor secretions, pyocyanin and elastase of *P. aeruginosa* PAO1 (Ng et al., 2011). The variant enzyme *Sso*Pox-W263I was further shown to significantly reduce the virulence of 51 clinical isolates of *P. aeruginosa* from diabetic foot ulcerations and kept its efficiency toward PAO1 after immobilization into polyurethane coating via glutaraldehyde crosslinking (Guendouze et al., 2017). The *in vivo* use of this variant was also reported through intratracheal administration and drastically enhanced the survival rate in a rat pneumonia model infected by *P. aeruginosa* PAO1 (Hraiech et al., 2014).

In addition to the studies using AHL-based QS quenchers, a recent report described the use of the AI-2 processing kinase LsrK. This enzyme was attached to a capsule of biological polymers chitosan and alginate supplemented with ATP substrate and reduced AI-2 mediated QS (Rhoads et al., 2017).

QQ-based devices have raised special attention considering that they could prevent HAIs by limiting bacterial virulence and biofilm formation. However, further efforts have to be dedicated to validate the proof of concepts *in vivo* and in clinical phases. The efficacy of these devices has to be demonstrated not only in model bacterial strains but also on genetically and phenotypically diverse clinical isolates. Although the development of medical devices is less constrained than for drugs, further regulatory concerns have to be considered to confirm the potential of the techniques for therapeutic applications. Nevertheless, the wide spectrum of both QSI and QQE as well as the numerous examples of their medical relevance would pave the way to the emergence of innovative devices.

## Conclusions and perspectives

Over the past 15 years, many studies have demonstrated that QQ molecules and QQ approaches have great potential as anti-infective agents against a broad range of bacteria. This is evidenced by the numerous studies demonstrating the benefit of these approaches in functionalizing medical devices. To date, little is known about potential resistance mechanisms that bacteria could develop to overcome QQ (Defoirdt et al., 2010; García-Contreras et al., 2013). The apparition of resistance phenomenon results from the natural process of evolution in a context of selection pressure which favors the growth of resistant strains. This is the case for antibiotics that apply high selection pressure, through growth inhibition, for sensitive strains (Davies and Davies, 2010). If some QSIs such as azithromycin lead to severe growth inhibition, others have only moderate or no effect on growth rate (García-Contreras et al., 2015). Apparition of QQ resistant bacteria is possible but its rate might be slower as compared to antibiotic resistance and will depend on the type of QQ (QSIs or QQEs) and its impact on bacterial growth (García-Contreras et al., 2016). QQ resistant strains have already been reported either from laboratory experiments or from clinical samples, notably strains with lower uptake or higher efflux of QSIs (Maeda et al., 2012). Nevertheless, little is known on how QQ resistant strains would over grow QQ sensitive strains nor on how the population would evolve. Most studies performed to address this question were performed *in vitro* and using QS mutants or QSIs, the results obtained so far were not consistent (Griffin et al., 2004; Gerdt and Blackwell, 2014; García-Contreras et al., 2016). To limit QQ resistance, QQ agents should be carefully chosen to keep growth deleterious effects minimal. Many QSIs have toxic activities and need to enter the cells in order to be active. QQ enzymes may represent ideal candidates combining a minimal, if any, selection pressure and potent inhibitory effects on biofilm formation and virulence (Guendouze et al., 2017). Further studies involving QQ enzymes should be performed to evaluate potential QQ resistance mechanisms using QQ enzymes. Nonetheless, QQ is a promising strategy to extend the therapeutic arsenal available to treat bacterial infections in complement to classical antimicrobial agents and antibiotics or reemerging bacteriophages.

The broad effect of QS on the physiology of bacteria shows that QQ would be an appropriate strategy not only to reducing bacterial virulence but also in terms of restoring antibiotic tolerance by decreasing biofilm formation and in terms of decreasing bacterial phage resistance, paving the way for future combination therapies.

Remarkably, the disruption of bacterial signaling, a communication system central to microbial communities (McFall-Ngai et al., 2013), has implications that go beyond the single bacteria physiology. Indeed, the gut microbiota of fishes fed with probiotic bacteria, producing QQ enzymes, was modified and the population of pathogenic *Aeromonas hydrophila* was reduced (Zhou et al., 2016). In another approach, a recent study showed the ability of *E. coli* overproducing AI-2 to counter the impact of streptomycin-induced gut dysbiosis potentially underlining the role of quorum sensing in the context of complex microbiota (Thompson et al., 2015). More studies are needed to delineate the effects induced by QQ strategies at both the single bacterial species level and in the context of communities. Future investigations will determine the breadth of the action of QQ molecules and their potential in being used as therapy, combination therapy and as coating agents in medical devices.

## Author contributions

BR, SM, LP, ME, EC, and DD: conceived and designed the work; BR, SM, LP, and DD: performed survey and drafted the paper; BR, SM, LP, ME, EC, and DD: critically revised the manuscript. All authors read and approved the final manuscript.

### Conflict of interest statement

ME and EC have a patent WO2014167140 A1 licensed to Gene&GreenTK. LP, DD, and EC report personal fees from Gene&GreenTK during the conduct of the study. The other authors declare that the research was conducted in the absence of any commercial or financial relationships that could be construed as a potential conflict of interest. The handling editor declared a past co-authorship with the authors ME, EC, and DD.
